# Radiation Therapy for Cutaneous Blastic Plasmacytoid Dendritic Cell Neoplasm: A Case Report and Review of the Literature

**DOI:** 10.3390/curroncol31110524

**Published:** 2024-11-13

**Authors:** Masashi Taka, Shinichiro Toyoshima, Shigeyuki Takamatsu, Satoshi Kobayashi

**Affiliations:** 1 Department of Radiotherapy, Toyama Prefectural Central Hospital, 2-2-78, Nishinagae, Toyama City 930-8550, Japan; shin@kem.biglobe.ne.jp; 2 Department of Radiology, Graduate School of Medical Sciences, Kanazawa University, 13-1, Takara-machi, Kanazawa City 920-8641, Japan; shigerad@staff.kanazawa-u.ac.jp (S.T.); satoshik@staff.kanazawa-u.ac.jp (S.K.)

**Keywords:** blastic plasmacytoid dendritic cell neoplasm, hematologic malignancy, radiation therapy, cutaneous lesions, dose fractionation, treatment protocol

## Abstract

Blastic plasmacytoid dendritic cell neoplasm (BPDCN) is a clinically aggressive hematologic malignancy derived from plasmacytoid dendritic cells. It commonly presents as cutaneous lesions. To date, no standard treatment protocol for BPDCN exists. Traditionally treated similarly to acute leukemia or lymphoma, its prognosis remains poor. Radiation therapy is employed for isolated skin lesions, for patients that are ineligible for chemotherapy due to age or comorbidities and for post-chemotherapy recurrence. However, very limited reports are available on radiotherapy for BPDCN. We present a case involving a 94-year-old BPDCN patient treated with radiation therapy, highlighting an atypical situation of two separate radiotherapy sessions with different dosages for isolated skin lesions. Initially, 45 Gy was administered in 15 fractions (45 Gy/15 Fr), followed by a second session of 30 Gy in 10 fractions (30 Gy/10 Fr) after disease recurrence. This case is unique in detailing radiation therapy for the exceedingly rare BPDCN, particularly dose fractionation. The findings indicate that 45 Gy/15 Fr can provide adequate local control, while even a lower dose of 30 Gy/10 Fr may be effective. This case report contributes to the limited literature by proposing potential therapeutic approaches and dosage guidelines to refine future BPDCN treatment protocols.

## 1. Introduction

Blastic plasmacytoid dendritic cell neoplasm (BPDCN) is a clinically aggressive hematologic malignancy derived from plasmacytoid dendritic cells (PDCs). It predominantly presents as cutaneous lesions, with potential bone marrow involvement and leukemic spread. The disease concept of BPDCN was established by the revised World Health Organization classification in 2008 [1]. As a rare entity, BPDCN accounts for approximately 0.5% of hematologic cancers [2]. Over 90% of patients initially exhibit skin lesions, including nodules, erythematous plaques, and bruise-like patches [3,4]. Suspected BPDCN cases, particularly those presenting with brown to violaceous bruise-like lesions, plaques, or tumors, warrant a skin biopsy for a definitive diagnosis. Diagnostic hallmarks include blastic or plasmacytoid morphology and PDC-specific expression of CD4, CD56, and CD123 [5]. Differential diagnoses include cutaneous malignancies and various leukemias. Reactive conditions like Kikuchi disease may also present with mature pDC accumulations.

To date, no consensus on a standard treatment for BPDCN exists. Historically treated akin to acute leukemia or lymphoma, BPDCN’s prognosis remains dire. Recent advancements include the development of tagraxofusp, a CD123-targeted cytotoxin composed of human interleukin-3 fused to truncated diphtheria toxin [2].

Radiation therapy is reserved for patients with isolated skin lesions, those ineligible for chemotherapy due to age or comorbidities, and for relapses post chemotherapy. Despite its use, detailed reports on radiotherapy dose fractionation for BPDCN are scarce, leaving its therapeutic efficacy and optimal dosages uncertain.

In a recent case, we administered radiotherapy to a newly diagnosed BPDCN skin lesion. Given the patient’s prognosis, age, and comorbidities, we opted for palliative radiotherapy aimed at aesthetic improvement rather than curative chemotherapy. Recurrent lesions were initially planned to receive a similar radiation dose; however, due to the patient’s deteriorating condition, a reduced dose was administered. This report details the unique course of receiving two distinct radiotherapy doses, discusses optimal dose fractionation, reviews the existing literature, and contributes valuable insights to medical knowledge.

## 2. Case Presentation

**Figure 1:**
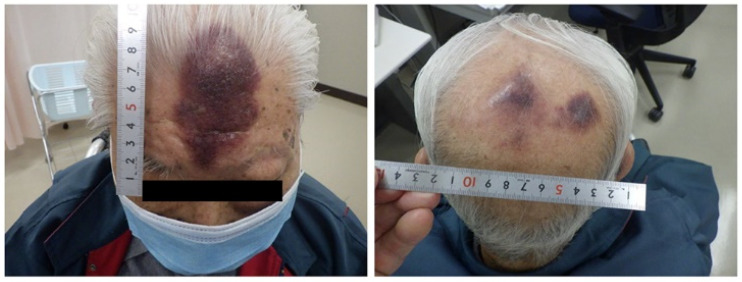
Macroscopic findings at the initial examination; a purple nodular lesion measuring 7.5 × 5 cm^2^ was noted on the frontal skin. Additionally, a purple “bruise-like” infiltrative spot, measuring 7 × 4 cm^2^, was present on the occipital skin.

A 94-year-old male patient with an Eastern Cooperative Oncology Group performance status (ECOG-PS) of 3, who had been contending with renal pelvis and bladder cancer for approximately 5 years and had been under the best supportive care from a urologist, developed a bruise-like rash on his forehead without any clear cause 4 months ago. This rash appeared and progressively enlarged over a period of 4 months. The initial clinical examination revealed a dome-shaped, dark purple, firm mass measuring 8 × 4 cm^2^ on the forehead, and there were also multiple pale dark skin eruptions with infiltration observed on the crown (Figure 1). No superficial lymph nodes were palpable. The clinician initially suspected hemangiosarcoma and proceeded to perform a skin biopsy from the lesions on the forehead. The histological examination demonstrated a diffuse proliferation of atypical cells extending from the dermis to the subcutaneous tissue. Immunohistochemical staining indicated that these atypical cells were diffusely and strongly positive for CD4, CD56, and CD123 and negative for CD3, CD34, and lysozyme. These findings led to the diagnosis of BPDCN.

**Figure 2:**
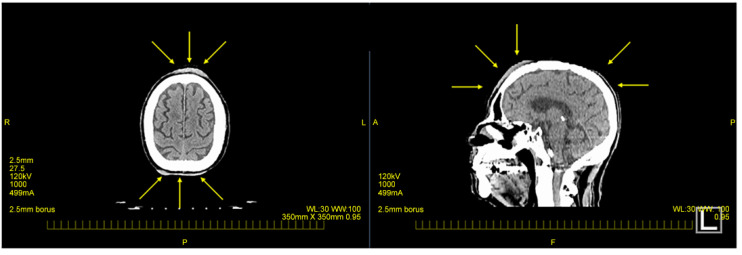
Imaging findings during the initial examination; CT imaging reveals thickening of the skin on the front and back of the head (yellow arrow).

Subsequently, a CT scan conducted to investigate systemic metastases revealed a thickening of the skin on the back of the head (Figure 2). However, no other significant distant lesions, including those in lymph nodes, were detected.

Blood tests showed no cytopenia or abnormal cells in the peripheral blood, but they did indicate severe renal dysfunction, attributed to underlying conditions such as bladder cancer and renal pelvis cancer. While induction chemotherapy might have been an option for a medically fit adult with a favorable ECOG-PS and without serious comorbidities, it was deemed unsuitable for this patient due to his ECOG-PS score, advanced age, pre-existing underlying diseases, and severe renal impairment. The patient expressed concern over the aesthetic impact of the prominent frontal lesion and wished to reduce its size. Therefore, palliative radiotherapy was administered with the goal of improving aesthetics through the local control of the lesion.

The patient was immobilized with a custom thermoplastic mask in a supine position and simulated by CT with a 2.5 mm slice thickness. Because the head lesion was not flat and reproducibility could not be guaranteed, no bolus was used. All simulation data were imported into the Eclipse^®^ treatment planning system (Varian Medical Systems, Inc., Palo Alto, CA, USA). The gross tumor volume (GTV) was defined as all known gross diseases according to clinical information and CT. The clinical target volume (CTV) included the GTV with an additional margin of 0.5 cm, not exceeding the periosteum of the cranium. The PTV was defined as the CTV plus a 0.5 cm margin.

**Figure 3:**
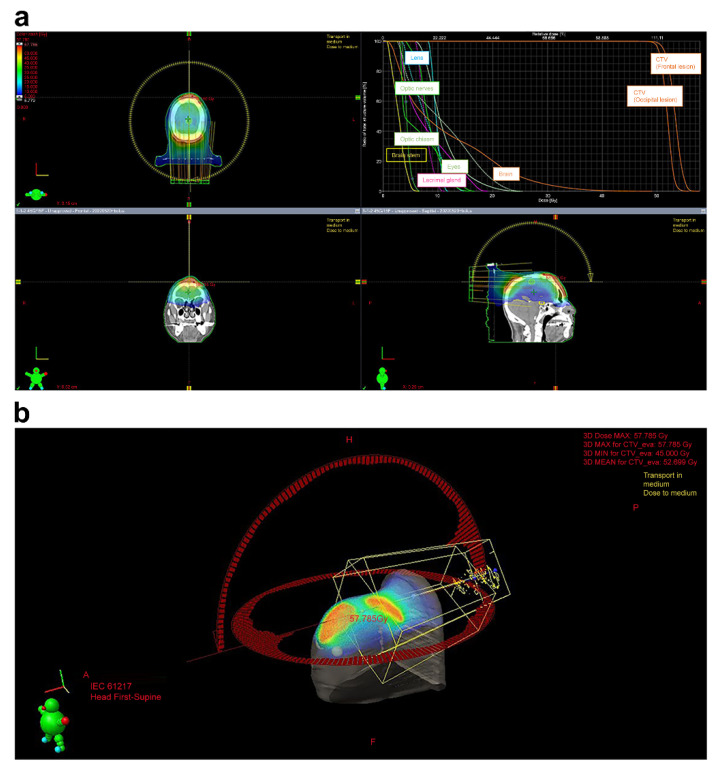
(**a**). Treatment plan; (**b**). The treatment plan was devised using the Eclipse treatment planning system. The VMAT plan utilized a 6 MV X-ray beam energy. The total radiation dose was prescribed as 45 Gy, delivered in 3 Gy per fraction. The plan was optimized to ensure target coverage with 100% (D100%) of the CTV.

The OAR included the spinal cord, brain, brainstem, chiasm, optic nerves, eyes, lenses, parotid glands, and inner ear. The planning organs at risk volume (PRV) were also used. The spinal cord, chiasm, optic nerves, and brainstem PRV were defined as the corresponding structures plus three-dimensional 0.3 cm margins, respectively. The VMAT plan utilized 6 MV X-ray beams. VMAT was used because there were multiple lesions on the head (which is not flat) and they could be treated simultaneously. The total radiation dose was 45 Gy at 3 Gy per fraction. Such a dose division corresponds to 48.75 Gy in terms of EQD2 when α/β = 10 Gy, which is a sufficient dose for aggressive lymphomas. This dose fractionation was chosen because it is sufficient for aggressive lymphoma, shortens the treatment period, and reduces the burden on the patient compared to 2 Gy per fraction. The plans were optimized to achieve 100% (D100%) coverage of the CTV (Figure 3a,b). The maximum and minimum doses to the skin on the CTV were approximately 120% (54 Gy) and 100% (45 Gy) of the prescribed dose, respectively.

**Figure 4:**
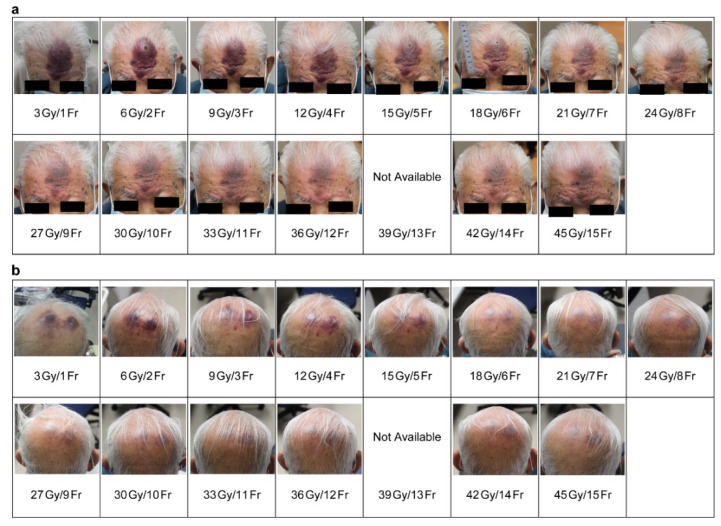
Progress during treatment ((**a**). frontal and (**b**). occipital lesions).

From around the sixth treatment, the raised lesions became less noticeable, and the color tone began to fade. At the end of the treatment, only grade 1 dermatitis was observed (Figure 4a,b). No acute adverse reactions of grade 2 or higher associated with the radiation therapy occurred.

Regarding the changes observed during irradiation, the raised skin lesions became less prominent, and their coloration started to fade after the sixth session of irradiation, likely because of high radiation sensitivity. By the 10th session, dermatitis was noticeable, and by the 15th session, the demarcation between the dermatitis and the lesion became partially indistinct, implying that the lesion had enlarged.

**Figure 5:**
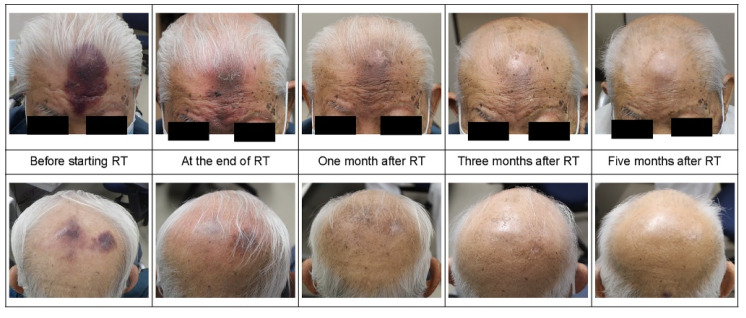
After irradiation changes at the 1-month follow-up after irradiation completion, the dermatitis was resolved, lesion area was notably reduced, and the lesion coloration was faded. Subsequently, the lesions continued to diminish, leaving only mild pigmentation 5 months after irradiation.

Further, 1 month after the end of the treatment, the dermatitis had disappeared, the lesions had clearly shrunk, and the color tone had faded. Three months after the end of the treatment, the lesions had almost disappeared, leaving only mild pigmentation (Figure 5).

**Figure 6:**
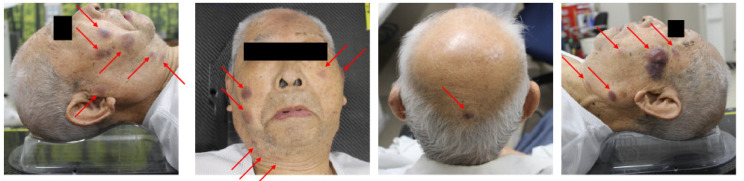
Macroscopic findings at ectopic recurrence (5 months after RT); numerous dark red, raised lesions emerged outside the irradiated field, indicating an ectopic recurrence of blastic plasmacytoid dendritic cell neoplasm (BPDCN).

**Figure 7:**
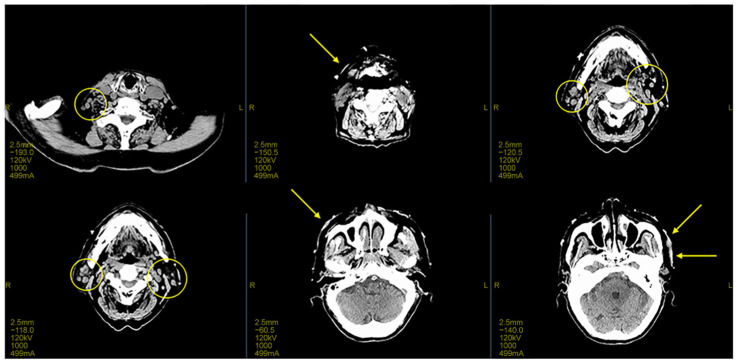
Imaging findings at ectopic recurrence; CT imaging during the ectopic recurrence showed skin thickening and enlarged lymph nodes (yellow arrow).

However, during the 5-month follow-up after treatment, new lesions appeared in untreated areas while the treated lesions remained controlled (Figure 6). A CT revealed a thickening of the skin on the back of the head as well as enlarged lymph nodes (Figure 7).

**Figure 8:**
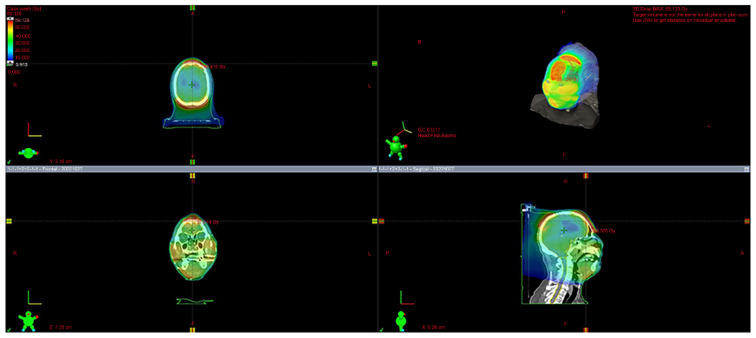
Cumulative dose diagram for initial irradiation and reirradiation; this is a dose distribution diagram that combines the initial irradiation of 45 Gy/15 Fr with reirradiation of 30 Gy/10 Fr.

A treatment plan of 45 Gy/15 Fr was similarly prepared for the new lesions, and treatment was started. However, it became difficult to continue the treatment due to the exacerbation of the bladder cancer and renal pelvis cancer, which were part of the patient’s medical history, and the treatment was terminated with 30 Gy/10 Fr (Figure 8).

**Figure 9:**
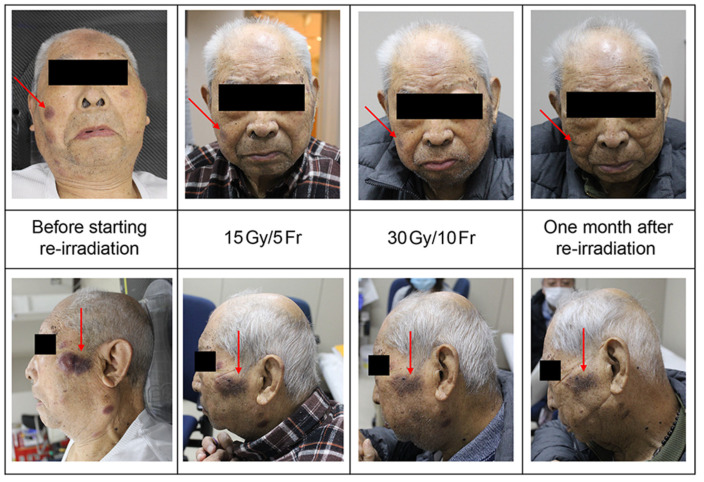
Changes during and after irradiation of recurrent lesions.

At the subsequent follow-up, there was no obvious increase in the ectopic recurrent lesions, and the color tone had slightly faded. The initial lesions remained in complete remission (Figure 9). Thereafter, his general condition deteriorated, and he died 9 months after the initial treatment.

Initially, there was an intention to treat the patient with 45 Gy/15 Fr as in the initial therapy; however, because of the declining overall health of the patient due to deteriorating renal pelvic and bladder cancer, irradiation was discontinued after administering 30 Gy/10 Fr. The sole adverse event observed at this juncture was very mild dermatitis. Follow-up was possible only for the first month thereafter; however, no considerable increase in the lesions was noted, and its coloration appeared to be fading. In a series of treatments, the irradiation field included the targeted skin and parts of the oral mucosa, salivary glands, or eyes; however, there were no adverse events such as stomatitis, xerostomia, and ocular disorders such as dry eyes or keratitis. Although the brain was partially included in the radiation field, no cognitive decline was observed for at least 9 months after the initial treatment.

## 3. Discussion

BPDCN is a rare, clinically aggressive hematologic malignancy that most commonly presents as bone marrow and skin lesions, with or without leukemic dissemination. The nomenclature for this entity has evolved over the years as our understanding of its underlying biology has improved. Initially described in 1995 as an acute agranular CD4-positive natural killer (NK) cell leukemia [6], it was later referred to as “blastic NK cell lymphoma” due to its blastic appearance and CD56 expression. Subsequently, the term “agranular CD4+CD56+ hematocutaneous neoplasm/tumor” was coined, reflecting the immunophenotype and skin lesion propensity. However, following the discovery that BPDCN originates from PDCs (type 2 dendritic cells), the 2008 World Health Organization classification of hematopoietic tumors adopted the term “plasmacytoid dendritic cell neoplasm” to describe this entity [1], a nomenclature retained in the 2016 revision [7].

BPDCN is a rare hematological tumor with an unknown exact incidence. Estimating its precise incidence is challenging due to evolving nomenclature and previously undefined criteria before the 2008 World Health Organization classification system. BPDCN accounts for 0.5% of all blood cancers [2], less than 1% of acute leukemias [8], 0.76% of acute myeloid leukemias [9], 0.27% of non-Hodgkin’s lymphomas [9], and 0.7% of primary cutaneous lymphoma [10]. However, these figures may be underestimations from cutaneous lymphoma registries, as a small yet significant number of patients present without skin involvement [11].

BPDCN occurs across all races and geographic locations. However, data on incidence variation by ethnicity or region are scarce. BPDCN has been reported in all age groups but is most prevalent in adults, with a majority of the patients being elderly. The median age at diagnosis ranges from 65 to 69 years [3,4,10]. The male-to-female ratio is approximately 2 to 3:1, with a slight male predominance [3,4,10]. BPDCN originates from bone marrow-derived quiescent plasmacytoid dendritic cell (type 2 dendritic cell) precursors [10,12,13,14].

Functional inactivation of TET2 is observed in 70–80% of BPDCN cases, often involving biallelic TET2 mutations [15,16,17]. It is believed that clonal TET2-deficient plasmacytoid dendritic cell progenitors migrate from the bone marrow to the skin and undergo a malignant transformation in response to ultraviolet (UV) damage [15]. Mutations in RAS, copy number loss of tumor suppressor genes (e.g., CDKN2A, SETD2, TP53), aberrant signaling from the E-box transcription factor TCF4, and/or overexpression of FLT3, HES6, and RUNX2 are implicated in the malignant transformation to BPDCN [18,19,20]. Additionally, studies have indicated a dependency on BCL2 expression, suggesting a potential sensitivity to the BCL2 inhibitor venetoclax [20].

Most BPDCN patients present with bone marrow and skin involvement, with or without leukemic dissemination [3]. One-third of cases show no signs of extracutaneous disease [4], while a small number present with leukemia and an absence of skin lesions [21,22]. Skin manifestations include brown to purple bruise-like lesions, plaques, or tumors that may be solitary or widespread [3]. The majority of patients exhibit cytopenia, lymphadenopathy, and/or splenomegaly [3,11], and liver involvement is more common in those with extensive bone marrow involvement [3]. Reports also include the involvement of the tonsils, paranasal sinuses, lungs, eyes, central nervous system (CNS), and paravertebral regions [3,11,22].

A retrospective series of 90 patients with skin lesions indicated that the clinical symptoms ranged from one or two skin nodules or tumors to disseminated skin spread [3]. Most patients exhibit brown or purple nodular lesions (73%), “bruise-like” brown to purple infiltrative macules (12%), and these can be categorized into the following three skin conditions: disseminated, solitary, and mixed lesions (14%) [4].

Approximately half of the patients initially present with focal nodular disease, consisting of one or two nodules, while 27% have multiple nodules affecting one or two areas [3]. The most common sites for local involvement are the face or scalp (20%), lower extremities (11%), trunk (9%), and upper extremities (7%). Subsequent evaluations reveal bone marrow, lymph node, or blood involvement in 61% of cases [3], with CNS involvement in 11%. About one-third show no evidence of nonskin disease [3].

Case reports also detail patients presenting with leukemia symptoms despite lacking skin disease [22]. These patients exhibit abnormal circulating “lymphoid/monocytoid” cells with or without leukocytosis, anemia, thrombocytopenia, hepatosplenomegaly, and lymphadenopathy.

Diagnostic criteria are stipulated by the World Health Organization Classification of Myeloid Tumors, 5th Edition (WHO5) [5] and the International Consensus Classification [23].

Histologically, BPDCN is characterized by a diffuse, monomorphic infiltrate of medium-sized blasts resembling lymphoblasts or myeloblasts [24]. The nucleus typically has an irregular outline with fine chromatin and one or more small nucleoli. The cytoplasm is generally scanty, gray-blue, and agranular, with a variable number of mitoses.

The immunophenotype should test positive for CD123, CD4, and/or CD56, along with at least one of the plasmacytoid dendritic cell (pDC) markers TCF4, TCL1, CD303 or CD304, via multicolor flow cytometry and/or immunohistochemistry. Typically, CD3, CD14, CD19, CD34, lysozyme, and myeloperoxidase are negative. Immunophenotyping may also be performed if three or more PDCs markers are expressed and all expected negative markers are absent [5].

The differential diagnosis for BPDCN includes malignancies with cutaneous manifestations and various leukemia such as acute myeloid leukemia, chronic myelomonocytic leukemia, mature pDC cell proliferation, nasal-type extranodal natural killer/T-cell lymphoma, subcutaneous panniculitis-like T-cell lymphoma, and cutaneous T-cell lymphoma. Accumulations of mature PDCs can also occur in some reactive conditions, like Kikuchi disease.

Optimal management strategies for BPDCN remain undefined. Conventional treatments modeled after those for acute leukemia and lymphoma have been largely unsuccessful. Patients undergoing allogeneic or autologous stem cell transplantation during the first remission often fare well; however, conventional chemotherapy is linked with early mortality rates of 17% to 26% and high relapse rates [2].

Nearly all cases of BPDCN exhibit the overexpression of interleukin-3 receptor subunit alpha (IL3RA or CD123) [25,26,27]. Tagraxofusp, a CD123-directed cytotoxin that fuses recombinant human interleukin-3 with truncated diphtheria toxin, has shown clinical benefits in adult patients with untreated or relapsed BPDCN [2].

The risk of recurrence is high, and the prognosis remains poor due to CNS involvement in BPDCN. Outcomes are most closely associated with age; a systematic review by Kim MJ et al., comparing 74 children to 283 adults (19 years of age and older) with BPDCN, reported more favorable outcomes in children [28]. Children had higher rates of complete remission (86 versus 52%, *p* < 0.01) and a longer mean time to death/relapse (12.0 ± 15.4 vs. 6.8 ± 6.4 months, *p* = 1.0).

In adults, being over 60 years old is linked to worse outcomes [28,29]. There seems to be no difference in the outcomes between patients presenting with skin-only disease and those with systemic disease. Even those with localized disease tend to have a progressive course and a poor prognosis [30]. MYC rearrangements have been associated with adverse outcomes [30,31,32,33], although the prognostic significance of other genetic features has not been clearly demonstrated.

Local radiation therapy has been utilized for patients with isolated skin lesions, particularly for those who are not suitable candidates for chemotherapy due to comorbidities or advanced age and for patients who have experienced a relapse after systemic chemotherapy [34].

However, reports on radiotherapy for BPDCN are scarce, with even fewer detailing radiation dose or fractionation. To our knowledge, doses ranging from 28.8 to 51 Gy (median 36 Gy) have been employed (Table 1) [9,35,36,37,38,39,40,41,42,43], but there are instances where CR occurred at doses around 27 Gy [37], suggesting that BPDCN may be highly radiosensitive, similar to malignant lymphoma.

In this particular case, the patient’s poor prognosis was influenced by age and an ECOG-PS score of 3, along with complications from bladder and kidney cancers and severe renal impairment. Consequently, no chemotherapy was administered. The presence of a prominent lesion in the frontal region posed an aesthetic concern for the patient, who requested a reduction in the lesion; therefore, palliative radiotherapy aimed at improving aesthetics through local control was performed. In the absence of clear guidelines, we believe this approach is one of the correct options.

For the dose fractionation tailored to this case, the initial lesion received treatment with 45 Gy/15 Fr. This dose division equates to an EQD2 of 48.75 Gy when α/β = 10 Gy, which is considered an adequate dose for aggressive lymphomas. The initial lesions treated with this regimen had almost disappeared approximately 3 months posttreatment, and no evident local recurrence was noted for at least 7 months posttreatment, signifying that 45 Gy/15 Fr was suitably effective for local control.

In terms of adverse events, grade 1 dermatitis and hair loss were noted; however, no severe adverse events were reported.

Even when considering reports from various sources, the dose division of 45 Gy/15 Fr appears to be an ample dose for the purpose of local control. For the ectopic recurrent lesion, due to a decline in general health caused by other illnesses, irradiation was unexpectedly concluded at 30 Gy/10 Fr. This dosage is equivalent to 32.5 Gy in EQD2 terms. Since his demise occurred shortly after, medium- to long-term follow-up could not be conducted; however, at least 1 month posttreatment for the ectopic recurrent lesion, there was no noticeable growth, and the lesion’s coloration seemed to be fading. Family reports also indicated that irradiation at 30Gy/10Fr alone was effective [9,37], suggesting that a certain level of local control might be anticipated with this dosage.

## 4. Conclusions

Here, we report the progression of a case where radiation therapy was unexpectedly administered twice with varying dose fractions for a BPDCN skin lesion. This constitutes the first detailed account of radiotherapy for the exceedingly rare BPDCN, inclusive of dose fractionation. Drawing from our experience and the literature, we believe that a dose fractionation of 45 Gy/15 Fr will ensure adequate local control. It was also suggested that a certain degree of local control can be expected with a dose fractionation of 30 Gy/10 Fr.

When considering local control in cases with a long-term prognosis, full consideration should be given to the dose fractionation of 45 Gy/15 Fr. Conversely, even in instances where a long-term prognosis is not anticipated, selecting 30 Gy/10 Fr may be viable as it can confer some level of local control. However, it is important to acknowledge that this report pertains to a single case, thereby imposing limitations on extrapolating these findings to all BPDCN patients. The accumulation of additional cases, continued research, and further validation are imperative. We hope that this report will aid in determining the appropriate dose fractionation for radiotherapy in BPDCN cases.

## Figures and Tables

**Table 1:** Radiotherapy literature for blastic plasmacytoid dendritic cell neoplasm.

Author	Age (years)/Sex	Treatment Site	Combination Therapy	Dose Fractionation	Treatment Effect	References
Jiang, Y.-L 2020	10/M	Scalp	Anti-CD123-CAR T-cell therapy	28.8 Gy/16 Fr	CR	[35]
Amitay-Laish, I 2017	38/M	Scalp	Hyper-CVAD	36 Gy	CR	[38]
Ishibashi, N 2015	77/M	Upper limb	None	30 Gy	PR	[9]
Heinicke, T 2015	62/F	Lower limb	CHOP	40 Gy	CR	[41]
Yu, G 2015	79/M	Back		50 Gy/25 Fr		[42]
Tsunoda, K 2012	74/M	Postauricular	None	27 Gy	CR	[37]
Miyashita, A 2011	77/M	Face	None	51 Gy	CR	[9]
Dohm, A 2011	32/M	Upper limb	CHOP	36 Gy	CR	[40]
Matsuo, T 2011	66/M	Chest, back, pharyx	Chemotherapy (details unknown)	30 Gy	Non-PD	[43]
Kaune, K.M 2009	66/F	Inner malleous	CHOP	34 Gy	PR	[39]
Fontaine, J 2009	60/F	Face	MTX, L-ASPDEX	40 Gy	CR	[36]
Our Case	90-/M	Scalp	None	45 Gy/15 Fr	CR	
		Face	None	30 Gy/10 Fr	SD	

Abbreviations: CR = complete response; PR = partial response; SD = stable disease; PD = progressive disease; Anti-CD123-CAR T-cell therapy = anti-CD123 chimeric antigen receptor (CAR) T-cell therapy; Hyper-CVAD = cyclophosphamide/vincristine/doxorubicin and dexamethasone; CHOP = cyclophosphamide/doxorubicin/vincristine and prednisone; MTX = methotrexate; L-ASP = l-asparaginase.

## Data Availability

No new data were created or analyzed in this study. Data sharing is not applicable to this article.

## References

[1] Facchetti F., Ungari M., Marocolo D., Lonardi S., Vermi W. Blastic plasmacytoid dendritic cell neoplasm. Proceedings of the7th Meeting New Insights in Hematology.

[2] Pemmaraju N., Lane A.A., Sweet K.L., Stein A.S., Vasu S., Blum W., Rizzieri D.A., Wang E.S., Duvic M., Sloan J.M. (2019). Tagraxofusp in blastic plasmacytoid dendritic-cell neoplasm. N. Engl. J. Med..

[3] Feuillard J., Jacob M.C., Valensi F., Maynadié M., Gressin R., Chaperot L., Arnoulet C., Brignole-Baudouin F., Drénou B., Duchayne E. (2002). Clinical and biologic features of CD4(+)CD56(+) malignancies. Blood.

[4] Julia F., Petrella T., Beylot-Barry M., Bagot M., Lipsker D., Machet L., Joly P., Dereure O., Wetterwald M., D’incan M. (2013). Blastic plasmacytoid dendritic cell neoplasm: Clinical features in 90 patients. Br. J. Dermatol..

[5] Khoury J.D., Solary E., Abla O., Akkari Y., Alaggio R., Apperley J.F., Bejar R., Berti E., Busque L., Chan J.K.C. (2022). The 5th edition of the World Health Organization Classification of Haematolymphoid Tumours: Myeloid and Histiocytic/Dendritic Neoplasms. Leukemia.

[6] Brody J.P., Allen S., Schulman P., Sun T., Chan W.C., Friedman H.D., Teichberg S., Koduru P., Cone R.W., Loughran T.P. (1995). Acute agranular CD4-positive natural killer cell leukemia. Comprehensive clinicopathologic studies including virologic and in vitro culture with inducing agents. Cancer.

[7] Arber D.A., Orazi A., Hasserjian R., Thiele J., Borowitz M.J., Le Beau M.M., Bloomfield C.D., Cazzola M., Vardiman J.W. (2016). The 2016 revision to the World Health Organization classification of myeloid neoplasms and acute leukemia. Blood.

[8] Bueno C., Almeida J., Marco L.P., Garcia J.R., Pablos D., Parreira J.M., Ramos A., Ruiz-Cabello F., Suarez-Vilela F., San Miguel J.F. (2004). Incidence and characteristics of CD4(+)/HLA DRhi dendritic cell malignancies. Haematologica.

[9] Ishibashi N., Maebayashi T., Aizawa T., Sakaguchi M., Abe O., Miura K., Hatta Y., Sugitani M. (2015). Radiation therapy for cutaneous blastic plasmacytoid dendritic cell neoplasm: A case report and review of the literature. Int. J. Clin. Exp. Med..

[10] Petrella T., Bagot M., Willemze R., Beylot-Barry M., Vergier B., Delaunay M., Meijer C.J.L.M., Courville P., Joly P., Grange F. (2005). Blastic NK-cell lymphomas (agranular CD4+CD56+ hematodermic neoplasms): A review. Am. J. Clin. Pathol..

[11] Pagano L., Valentini C.G., Pulsoni A., Fisogni S., Carluccio P., Mannelli F., Lunghi M., Pica G., Onida F., Cattaneo C. (2013). Blastic plasmacytoid dendritic cell neoplasm with leukemic presentation: An Italian multicenter study. Haematologica.

[12] Chaperot L., Bendriss N., Manches O., Gressin R., Maynadie M., Trimoreau F., Orfeuvre H., Corront B., Feuillard J., Sotto J.J. (2001). Identification of a leukemic counterpart of the plasmacytoid dendritic cells. Blood.

[13] Marafioti T., Paterson J.C., Ballabio E., Reichard K.K., Tedoldi S., Hollowood K., Dictor M., Hansmann M.-L., Pileri S.A., Dyer M.J. (2008). Novel markers of normal and neoplastic human plasmacytoid dendritic cells. Blood.

[14] Sapienza M.R., Fuligni F., Agostinelli C., Tripodo C., Righi S., Laginestra M.A., Pileri A., Mancini M., Rossi M., Ricci F. (2014). Molecular profiling of blastic plasmacytoid dendritic cell neoplasm reveals a unique pattern and suggests selective sensitivity to NF-kB pathway inhibition. Leukemia.

[15] Griffin G.K., Booth C.A.G., Togami K., Chung S.S., Ssozi D., Verga J.A., Bouyssou J.M., Lee Y.S., Shanmugam V., Hornick J.L. (2023). Ultraviolet radiation shapes dendritic cell leukaemia transformation in the skin. Nature.

[16] Khanlari M., Yin C.C., Takahashi K., Lachowiez C., Tang G., Loghavi S., Bah I., Wang W., Konoplev S., Medeiros L.J. (2022). Bone marrow clonal hematopoiesis is highly prevalent in blastic plasmacytoid dendritic cell neoplasm and frequently sharing a clonal origin in elderly patients. Leukemia.

[17] Togami K., Chung S.S., Madan V., Kenyon C.M., Cabal-Hierro L., Taylor J., Kim S.S., Griffin G.K., Ghandi M., Li J. (2022). Sex-Biased ZRSR2 mutations in myeloid malignancies impair plasmacytoid dendritic cell activation and apoptosis. Cancer Discov..

[18] Ceribelli M., Hou Z.E., Kelly P.N., Huang D.W., Wright G., Ganapathi K., Evbuomwan M.O., Pittaluga S., Shaffer A.L., Marcucci G. (2016). A druggable TCF4− and BRD4-dependent transcriptional network sustains malignancy in blastic plasmacytoid dendritic cell neoplasm. Cancer Cell.

[19] Dijkman R., Van Doorn R., Szuhai K., Willemze R., Vermeer M.H., Tensen C.P. (2007). Gene-expression profiling and array-based CGH classify CD4+CD56+ hematodermic neoplasm and cutaneous myelomonocytic leukemia as distinct disease entities. Blood.

[20] Montero J., Stephansky J., Cai T., Griffin G.K., Cabal-Hierro L., Togami K., Hogdal L.J., Galinsky I., Morgan E.A., Aster J.C. (2017). Blastic plasmacytoid dendritic cell neoplasm is dependent on BCL2 and sensitive to venetoclax. Cancer Discov..

[21] Rauh M.J., Rahman F., Good D., Silverman J., Brennan M.K., Dimov N., Liesveld J., Ryan D.H., Richard Burack W., Bennett J.M. (2012). Blastic plasmacytoid dendritic cell neoplasm with leukemic presentation, lacking cutaneous involvement: Case series and literature review. Leuk. Res..

[22] Martín-Martín L., Almeida J., Pomares H., González-Barca E., Bravo P., Giménez T., Heras C., Queizán J.-A., Pérez-Ceballos E., Martínez V. (2016). Blastic plasmacytoid dendritic cell neoplasm frequently shows occult central nervous system involvement at diagnosis and benefits from intrathecal therapy. Oncotarget.

[23] Arber D.A., Orazi A., Hasserjian R.P., Borowitz M.J., Calvo K.R., Kvasnicka H.M., Wang S.A., Bagg A., Barbui T., Branford S. (2022). International consensus classification of myeloid neoplasms and acute leukemias: Integrating morphologic, clinical, and genomic data. Blood.

[24] Campo E., Harris N.L., Jaffe E.S., Pileri S.A., Stein H., Thiele J., Swerdlow S.H. (2017). WHO classification of tumours of haematopoietic and lymphoid tissues. WHO Classification of Tumours.

[25] Jordan C.T., Upchurch D., Szilvassy S.J., Guzman M.L., Howard D.S., Pettigrew A.L., Meyerrose T., Rossi R., Grimes B., Rizzieri D.A. (2000). The interleukin-3 receptor alpha chain is a unique marker for human acute myelogenous leukemia stem cells. Leukemia.

[26] Han L., Qiu P., Zeng Z., Jorgensen J.L., Mak D.H., Burks J.K., Schober W., Mcqueen T.J., Cortes J., Tanner S.D. (2015). Single-cell mass cytometry reveals intracellular survival/proliferative signaling in FLT3-ITD-mutated AML stem/progenitor cells. Cytom. Part. A.

[27] Testa U., Pelosi E., Frankel A. (2014). CD 123 is a membrane biomarker and a therapeutic target in hematologic malignancies. Biomark. Res..

[28] Kim M.J.-M., Nasr A., Kabir B., De Nanassy J., Tang K., Menzies-Toman D., Johnston D., El Demellawy D. (2017). Pediatric blastic plasmacytoid dendritic cell neoplasm: A systematic literature review. J. Pediatr. Hematol. Oncol..

[29] Reimer P., Rüdiger T., Kraemer D., Kunzmann V., Weissinger F., Zettl A., Konrad Müller-Hermelink H., Wilhelm M. (2003). What is CD4+CD56+ malignancy and how should it be treated?. Bone Marrow Transpl..

[30] Taylor J., Haddadin M., Upadhyay V.A., Grussie E., Mehta-Shah N., Brunner A.M., Louissaint A., Lovitch S.B., Dogan A., Fathi A.T. (2019). Multicenter analysis of outcomes in blastic plasmacytoid dendritic cell neoplasm offers a pretargeted therapy benchmark. Blood.

[31] Boddu P.C., Wang S.A., Pemmaraju N., Tang Z., Hu S., Li S., Xu J., Medeiros L.J., Tang G. (2018). 8q24/MYC rearrangement is a recurrent cytogenetic abnormality in blastic plasmacytoid dendritic cell neoplasms. Leuk. Res..

[32] Sakamoto K., Katayama R., Asaka R., Sakata S., Baba S., Nakasone H., Koike S., Tsuyama N., Dobashi A., Sasaki M. (2018). Recurrent 8q24 rearrangement in blastic plasmacytoid dendritic cell neoplasm: Association with immunoblastoid cytomorphology, MYC expression, and drug response. Leukemia.

[33] Lezama S., Chisholm L., Carneal K.M., Nagy E., Cascio A., Yan M.J., Chang J., Cherry C.C., George A., Ohgami T.I. (2018). An analysis of blastic plasmacytoid dendritic cell neoplasm with translocations involving the MYC locus identifies t(6;8)(p21;q24) as a recurrent cytogenetic abnormality. Histopathology.

[34] Falcone U., Sibai H., Deotare U. (2016). A critical review of treatment modalities for blastic plasmacytoid dendritic cell neoplasm. Crit. Rev. Oncol. Hematol..

[35] Jiang Y.-L., Li Q., Yuan T., Jiang Y.-Y., Deng Q. (2020). Case report of anti-CD123 chimeric antigen receptor T-cell therapy followed by radiotherapy for a recurrence of blastic plasmacytoid dendritic cell neoplasm after allogeneic hematopoietic stem cell transplantation. Onco Targets Ther..

[36] Fontaine J., Thomas L., Balme B., Ronger-Savle S., Traullé C., Petrella T., Dalle S. (2009). Haematodermic CD4+CD56+ neoplasm: Complete remission after methotrexate-asparaginase treatment. Clin. Exp. Dermatol..

[37] Tsunoda K., Satoh T., Akasaka K., Ishikawa Y., Ishida Y., Masuda T., Akasaka T. (2012). Blastic plasmacytoid dendritic cell neoplasm: Report of two cases. J. Clin. Exp. Hematop..

[38] Amitay-Laish I., Sundram U., Hoppe R.T., Hodak E., Medeiros B.C., Kim Y.H. (2017). Localized skin-limited blastic plasmacytoid dendritic cell neoplasm: A subset with possible durable remission without transplantation. JAAD Case Rep..

[39] Kaune K.M., Baumgart M., Bertsch H.P., Mitteldorf C., Müller-Hermelink H.K., Haase D., Ghadimi B.M., Schön M.P., Neumann C. (2009). Solitary cutaneous nodule of blastic plasmacytoid dendritic cell neoplasm progressing to overt leukemia cutis after chemotherapy: Immunohistology and FISH analysis confirmed the diagnosis. Am. J. Dermatopathol..

[40] Dohm A., Hasenkamp J., Bertsch H.P., Maas J.H., Truemper L., Wulf G. (2011). Progression of a CD4+/CD56+ blastic plasmacytoid DC neoplasm after initiation of extracorporeal photopheresis in an allogeneic transplant recipient. Bone Marrow Transpl..

[41] Heinicke T., Hütten H., Kalinski T., Franke I., Bonnekoh B., Fischer T. (2015). Sustained remission of blastic plasmacytoid dendritic cell neoplasm after unrelated allogeneic stem cell transplantation—A single center experience. Ann. Hematol..

[42] Yu G., Wang W., Han Y., Liu J., Pan X., Qu G. (2015). Blastic plasmacytoid dendritic cell neoplasm presenting with a cutaneous tumor alone as the first symptom of onset: A case report and review of literature. Oncol. Lett..

[43] Matsuo T., Ichimura K., Tanaka T., Morizane S., Iwatsuki K., Eguchi M., Yoshino T. (2011). Bilateral conjunctival lesions in blastic plasmacytoid dendritic cell neoplasm. J. Clin. Exp. Hematop..

